# Oncocalyxone A Functions As an Anti-Glycation Agent In Vitro

**DOI:** 10.1371/journal.pone.0131222

**Published:** 2015-06-25

**Authors:** Ingrid Sofia Vieira de Melo, Aldenir Feitosa dos Santos, Telma Leda Gomes de Lemos, Marília Oliveira Fonseca Goulart, Antônio Euzébio Goulart Santana

**Affiliations:** 1 Instituto de Química e Biotecnologia, Universidade Federal de Alagoas. Endereço: Cidade universitária, BR 101 (km 14), Tabuleiro dos Martins, CEP, 57072–970, Maceió, AL, Brasil; 2 Departamento de agroindústria, Instituto Federal de Alagoas. Endereço: Conjunto Residencial Astolfo Lopes, s/n—Cidade Alta, CEP, 57820–000, Murici, AL, Brasil; 3 Departamento de Química, Universidade Estadual de Alagoas. Endereço: Av. Governador Luís Cavalcante, Alto do Cruzeiro, CEP, 57312–270, Arapiraca, AL, Brasil; 4 Departamento de Química Orgânica e Inorgânica, Universidade Federal do Ceará. Endereço: Av. Mister Hull, Campus do Pici, CEP, 60451–970, Fortaleza, CE, Brasil; Institut national de la santé et de la recherche médicale—Institut Cochin, FRANCE

## Abstract

Advanced glycation endproducts (AGE) are the result of post-translational changes to proteins, which ultimately compromise their structure and/or function. The identification of methods to prevent the formation of these compounds holds great promise in the development of alternative therapies for diseases such as diabetes. Plants used in traditional medicine are often rich sources of anti-glycation agents. Therefore, in this study, we investigated the anti-glycation activity of one such compound, Oncocalyxone A (Onco A). Using spectrofluorimetric techniques, we determined that Onco A inhibits AGE formation in a concentration-dependent manner. Its IC_50_ value (87.88 ± 3.08 μM) was almost two times lower than the standard anti-glycation compound aminoguanidine (184.68 ± 4.85 μM). The excellent anti-glycation activity of Onco A makes it an exciting candidate for the treatment of diseases associated with excessive accumulation of AGE. However, additional studies are necessary to identify its mechanism of action, as well as the *in vivo* response in suitable model organisms.

## Introduction

Glycation is a non-enzymatic protein modification that occurs when proteins react with sugar molecules and/or metabolized intermediates, such as glyoxal or methylgloxal. These reactions occur both *in vitro* and *in vivo*. The first step of the process, the Maillard reaction, leads to the irreversible formation of advanced glycation end products (AGE), which ultimately interact with proteins resulting in damage and degradation. The accumulation of AGE plays an important role in age-related disorders and heart failure due to diabetic complications [1].

AGE arise as the result of post-translational protein modifications [2], and these changes may promote protein-protein interactions through the formation of intra- and intermolecular bonds, ultimately resulting in dysfunctional protein structure and/or function. In the blood, significant accumulation of AGE alters protein dynamics, and this has been attributed to premature aging and the increasing incidence of chronic diseases [3]. Indeed, there is a large body of work indicating that nonenzymatic glycation is involved in other disorders as well, such as arthritis, atherosclerosis, chronic renal failure, Alzheimer's disease, nephropathy, neuropathy, and cataracts [4]. Therefore, controlling the formation of these harmful intermediaries via anti-glycation agents has far-reaching implications in the treatment of various diseases.

There have been several proposed mechanisms of action of these anti-glycation agents, including: (1) the elimination of reactive oxygen species (ROS) and reactive nitrogen species (RNS), mainly hydroxyl radicals and superoxide radical anions, to decrease oxidative stress and reduce the generation of carbonyl or dicarbonyl groups; (2) the elimination of Amadori products and Schiff bases by preventing the formation of carbonyl and dicarbonyl compounds; (3) the chelation of free metals as the formation of AGE are related to the presence of transition metal ions; (4) the inhibition of the formation of an Amadori product in the final stage of glycation; (5) by breaking protein crosslinks promoted by AGE; (6) the occlusion of cellular AGE receptors (RAGE), which can reduce oxidative stress and inflammation in biological systems, and ultimately, prevent the late stages of glycation. Among the mechanisms described above, the first four are documented for naturally occurring anti-glycation products, while the final two are more commonly attributed to therapeutic agents such as aspirin, aminoguanidine, and diethylenetriaminepentaacetic acid. Antioxidant compounds are of particular interest as candidates as anti-glycation agents, given that they inhibit the formation of naturally occurring AGE, providing a strong therapeutic prospect toward delaying and preventing diabetic complications. Still, the mechanisms that account for the inhibitory pathways described above are poorly understood [5].

ROS and RNS play important roles in the non-enzymatic glycation of proteins [6]. In recent years, several natural products have been characterized as having anti-glycation activity, and often this correlates with antioxidant properties [7]. There are reports that many food products containing antioxidant compounds are able to eliminate ROS and RNS generated during glycation, as well as prevent the auto-oxidation of reducing sugars and Amadori products, and both are favorable ways for the inhibition of AGE [8].

The characterization of biological compounds identified in plant extracts is still in its infancy, due in large part to the significant biodiversity that exists, especially among Brazil’s fauna. The isolation and possible development of an anti-glycation biocompound from these plants would be a highly relevant biotechnological discovery with significant importance for individuals worldwide [9].

**Fig 1:**
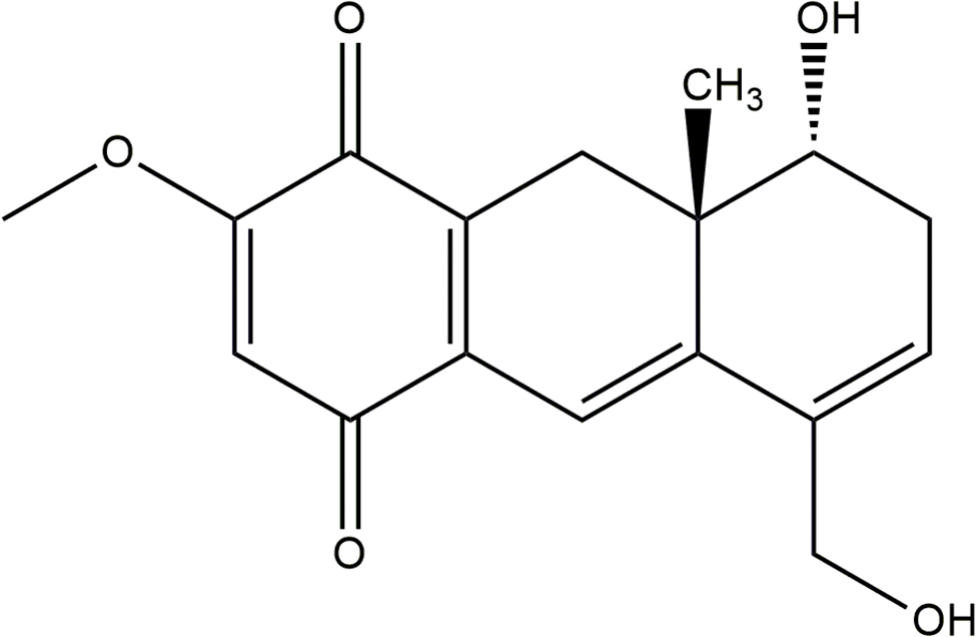
Chemical structure of Oncocalyxone A.

The *Auxemma oncocalyx* Taub (Boraginaceae) is a plant native to Brazil’s Northeast region, and it is quite common in the state of Ceará. Popularly known as “pau branco,” it is used locally as an astringent and for the treatment of wounds [10]. Chemical studies of *A*. *oncocalyx* Taub resulted in the isolation and characterization of various quinones. The first compound isolated from the ethanolic extract was oncocalyxone A [rel-8α-hydroxy-5-hydroxymethyl-2-methoxy-8α, β-methyl-7, 8, 8a, 9-tetrahydro-1 4-anthracenedione] (Onco A; Fig 1), which is characterized as a red solid, and most abundant quinone in this fraction [11, 12].

Onco A exhibited various activities *in vivo*, including cytotoxic, analgesic and antioxidant functions, and inhibitory effects on inflammation, edema, platelet aggregation and tumorigenicity [10, 13, 14, 15]. Additionally Onco A is known to inhibit cell growth by damaging DNA [15,16]. Strikingly, [17] reported that Onco A isolated from leaves of *Prenanthes sarmentosus* exerted anti-diabetic effects in rats with alloxan-induced diabetes. The animals had significantly lower blood glucose levels in the treated rats than the controls and an overall increase in body weight.

With the above in mind, the objective of this study was to characterize the inhibition of AGE formation and potential anti-glycation activity of Onco A *in vitro*.

## Methods

*A*. *oncocalyx* Taub was collected at Pentencoste City in the state of Ceara, which is located in Northeast Brazil. The species was identified by Professor Afranio Gomes Fernandes from the Department of Biology of the Federal University of Ceara, where a voucher was deposited at the Prisco Bezerra Herbarium under the registration number 18459. OncoA was isolated as a deep red powder, mp 207–208°C, from the ethanol extract of the heartwood of *A*. *oncocalyx* as previously described [11]. The structure of this secondary metabolite was unambiguously established by spectrometric techniques such as IR, MS and a combination of 1D and 2D NMR methods. Its structural identity and purity (93.26%) were confirmed by thin layer chromatography (comparison with authentic sample), mixed melting point and 1H- and 13C-NMR spectral analysis [18].

To measure anti-glycation activity, solutions of bovine serum albumin (BSA 2 mg / mL, spectrophotometrically determined), glucose (200 mM) and fructose (200 mM) were first prepared in sodium phosphate monobasic monohydrate buffer (100 mM pH 7.4). All chemicals were purchased from Sigma Aldrich (Brazil).

This assay followed that described by Beaulieu et al. [9]. Initially the incubation media consisted of BSA (1 mg / mL), glucose (100 mM), fructose (100 mM) and either Onco A or aminoguanidine (solvent; 80% EtOH, 20% H_2_O) in 100 mM sodium phosphate buffer. Six different concentrations of Onco A (10.35; 41.39; 82.78; 165.56; 331.13; 662.25 μM) were analyzed to determine IC_50_. Once BSA was auto fluorescent, a negative control consisting of glucose (100 mM), fructose (100 mM), BSA (1 mg / mL) and vehicle (100 mM phosphate buffer), and a positive control containing aminoguanidine (3.52–452.38 μM), were prepared an assayed. The samples were incubated in the dark at 37°C with constant stirring for 7 days and the formation of AGE was quantified by spectrofluorimetry (λ_ex_ = 355 and λ_em_ = 440 nm) [5], The experiment was conducted in three independent replicates.

Fluorescence readings and controls were normalized to corresponding blanks to exclude baseline fluorescence. The fluorescence (F) corrected for the negative control (F_negative control_) and experimental treatments (F_experimental corrected_) were used to determine the percentage of inhibition of the formation of AGE using the following formula [9]:

$$\text{Inhibition Percentage}=\;\left({\text{F}}_{\text{negative control}}-\;{\text{F}}_{\text{experimental corrected}}/\;{\text{F}}_{\text{negative control}}\right)$$

The IC_50_ values, defined as the amount of compound (μM) necessary to reduce 50% of AGE formation as compared to the negative control, were determined using regression analysis.

## Results and Discussion

**Fig 2:**
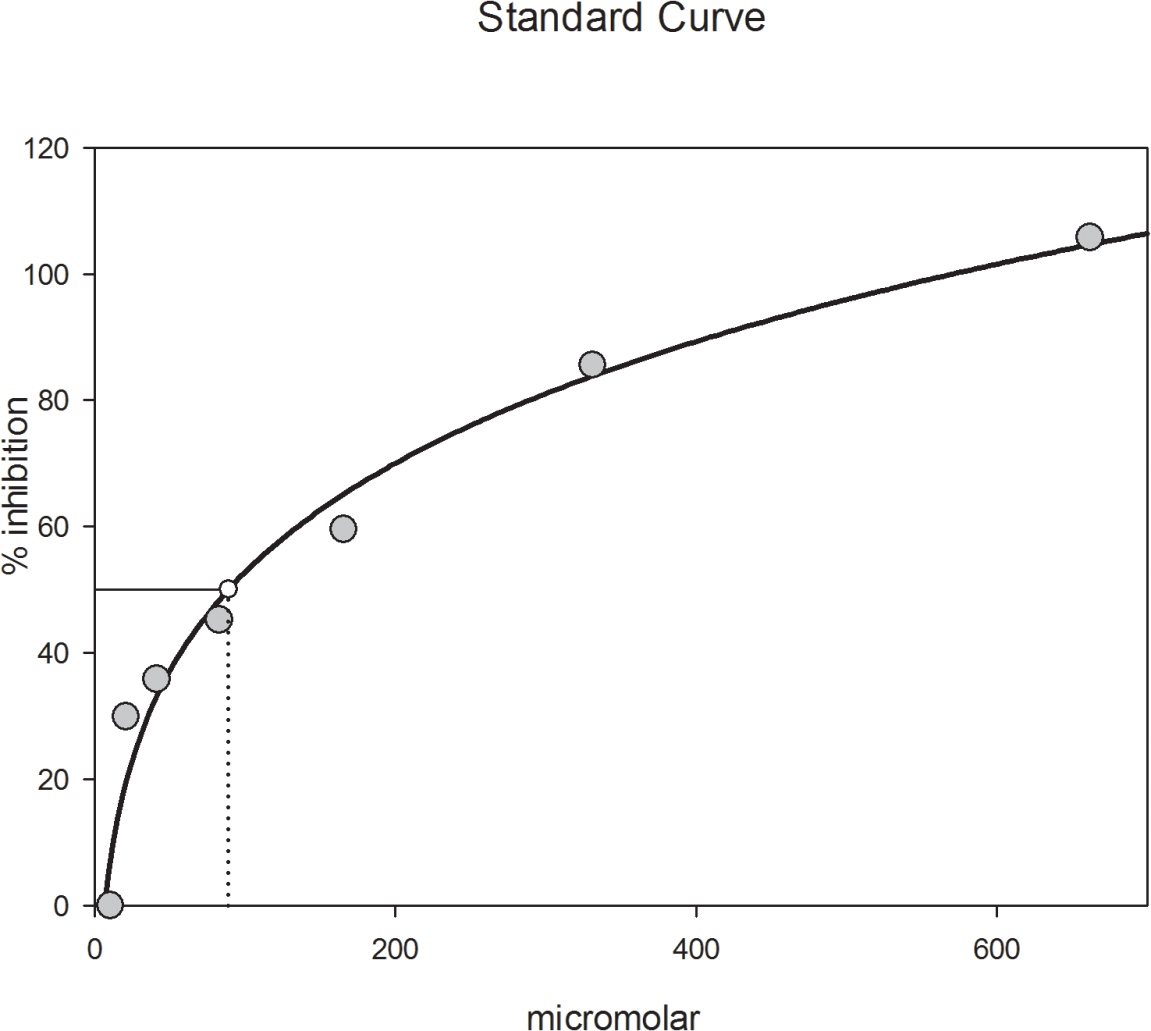
Dose-dependent inhibition of the fluorescent AGE formation by Onco A, via spectrofluorimetric method. *r* ^*2*^ 0.97.

Fig 2 shows dose response analysis for Onco A. Glycation was efficiently inhibited by Onco A in a concentration-dependent manner. The anti-glycation activity of the compound (IC_50_ = 87.88 ± 3.08 μM) was almost twice that of the current standard, aminoguanidine (IC_50_ = 184.68 ± 4.85 μM), reinforcing the excellent activity demonstrated by this quinone.

The anti-glycation activity observed in the present study is in agreement with the findings of Khan et al. [19]. While testing derivatives of *N*-aroyl-isatin, the group reported that of their 15 compounds tested, 6 displayed anti-glycation activity with IC_50_ values ranging from 18.01 to 171.3 μM. These values are notably lower than aminoguanidine (268.7 μM). Additionally, Khan et al. [20] analyzed 27 bis-Schiff bases derived from isatins and found three with excellent anti-glycation activity. These compounds had IC_50_ values in the range of 243.95–291.14 μM, which exceeded the standard used in their study, rutine (294.46 μM). In fact, these values were over two times higher than those obtained for Onco A, reinforcing again the significance of the anti-glycation activity reported here, Table 1.

**Table 1:** Anti-glycation activity of pure compounds.

References	Compounds	μM	Used method*
	Aminoguanidine	184.68 ± 4.85	BSA/Glucose/Fructose
	Oncocalyxone A	87.88 ± 3.08	
*Khan et al*., *2010* [19]	Aminoguanidine	268.7	BSA/Glucose
	1-Benzoyl-1H-indol-2,3 dione	18.01	
	1-(3-Methylbenzoyl)-1H-indol-2,3 dione	170.2	
	1-(4-Nitrobenzoyl)-1H-indol-2,3 dione	80.18	
	1-(3-Chlorobenzoyl)-1H-indol-2,3 dione	117.91	
	1-(4-Chlorobenzoyl)-1H-indol-2,3 dione	72.5	
	1-(2-Fluorobenzoyl)-1H-indol-2,3 dione	171.3	
*Khan et al*., *2009*[20]	Rutin	294.46	BSA/MGO**
	3,4-Dihidroxybenzaldehyde-N-(2-oxo-1,2-dihidro-3H-indol-3-ylidene) hidrazone	291.14	
	4-Nitrobenzaldehyde-N-(2-oxo-1,2-dihydro-3H-indol-3-ylidene) hidrazone	257.61	
	2-Nitrobenzaldehyde-N-(2-oxo-1,2-dihidro-3H-indol-3-ylidene) hidrazone	243.95	
*Choudhary et al*. *(2011)*[21]	Rutin	294.5	BSA/MGO**
	*Ethyl Heamatomate*	220.55	
*Magadula et al*., *2014* [22]	*Morelloflavona*	78.0	BSA/D-ribose
*Muhammad et al*., *2013* [23]	Rutin	294	BSA/Glucose/MGO
	*2*,*4-diidroxy-5-methoxy-cinnamic acid*	355	
*Park et al*., *2010* [24]	Aminoguanidine	479	BSA / Glucose
	2',4',5-triidroxy-[5''-(1,2-diidroxy-1-methylethyl)- Diidrofuran (2'',3'':7,8)]-(3*S*)-isoflavanone	20.6	
	2',4',5-triidroxy-[5''-(1,2-diidroxy-1-methylethyl)-diidrofuran(2'',3'':7,8)]-(3*R*)-isoflavanone	18.4	
	Catechin	5.4	

* Base components used to promote glycation.

** MGO Methylglioxal

Additionally, Onco A was found to be twice as effective as ethyl heamatommate, the most active compound (IC_50_ = 220.55 μM) isolated from *Parmotrema cooperi* by Choudhary et al. [21], and three times as effective as the standard compound, rutin (IC_50_ = 294.5 μM), used in their study. The authors reported that the presence of carbonyl and hydroxyl groups might be responsible for the anti-glycation activity of this series of compounds. Magadula et al. [22] found that morelloflavona, an isolated biflavonoid from *Garcinia volkensis*, was a very effective inhibitor of AGE formation with an IC_50_ a little lower than that of Onco A (78 μM), according to Table 1.

Muhammad et al. [23] discovered that a derivative of cinnamic acid, the 2,4-dihydroxy-5-methoxy-cinaminic acid, isolated from the plant *Viola betonicifolia* moderately inhibited the formation of AGE, with IC_50_ value of 355 μM. Note this is three times greater than the IC_50_ of Onco A (87.88 ± 3.08 μM). Park et al. [24] have reported that three compounds isolated from *Lespedeza maximowiczi* are actually more active than Onco A, with IC_50_ values ranging from 5.4–20.6 μM.

Regarding the molecular mechanism of Onco A, it is possible that its anti-glycation effects are via ROS inhibition, as this compound has been identified in previous antioxidant studies from Ferreira et al. [10]. The authors demonstrated that the quinone fraction of *A*. *oncocalyx*, consisting mainly of Onco A, has hepatoprotective effects in the CCl_4_-induced hepatotoxicity rat model, where both free radicals and lipid peroxidation are involved. They clarified that these hepatoprotective effects were mediated by the fraction’s antioxidant function, which was verified by surveying the plasma activity of glutamate-pyruvate transaminase (GTP) and glutamate-oxalate-transaminase (GOT). However, this may not be the sole anti-glycation mechanism presented by Onco A.

Importantly, Ferreira et al. [14] identified the anti-platelet aggregation activity of Onco A, which is associated with increased levels of cGMP-independent nitric oxide (NO) and blockade of the glycoprotein GP Ibα. Accumulation of AGE in tissues, and the corresponding dysfunction in platelet aggregation, may promote diabetic complications such as retinopathy, neuropathy, and renal dysfunction [25]. Yamagishi et al. [26] examined the effects of AGE on the production of prostacyclin (PGI2), an antithrombogenic prostanoid, and the inhibitor of endothelial plasminogen activator 1 (PAI-1). They found that increased accumulation of AGE promotes platelet aggregation and PAI-1 expression, while also inhibiting prostacyclin expression, in endothelial cells. This suggests that AGE have the ability to trigger platelet aggregation and fibrin stabilization, resulting in a predisposition to thrombogenesis, and ultimately, the development of diabetic vascular complications [26]. It appears likely that the inhibitory effects of Onco A on platelet aggregation are related to its anti-glycation activity, as there is an intimate association with excessive formation of AGE and the stimulation of platelet aggregates.

Thus, it is possible that the three major functions attributed to Onco A, antioxidant, anti-diabetic, anti-platelet aggregation are associated to the anti-glycation properties of the compound.

## Conclusion

Onco A demonstrated potent inhibitory effects on glucose/fructose induced protein glycation, and this superior activity suggests it may potentially serve as a viable treatment targeted at preventing diabetic complications, as well as slowing the onset and progression of other AGE-related diseases. However, these findings should be taken with some caution, given the moderate cytotoxicity of the compound.

Still, it seems reasonable to propose that the dramatic effects Onco A has on reactive oxygen and platelet aggregation are associated with the anti-glycation activity demonstrated in this study, although the signaling mechanisms remain poorly understood. For these reasons, future studies in model organisms are both appropriate and necessary to further understanding of the benefits of Onco A.

## Supporting Information

S1 File Database. (XLSX)
